# Cyanobacteria in wetlands of the industrialized Sambalpur District of India

**DOI:** 10.1186/2046-9063-9-14

**Published:** 2013-07-12

**Authors:** Pratibha Rani Deep, Shantanu Bhattacharyya, Binata Nayak

**Affiliations:** 1 CyanoLab, School Of Life Sciences, Sambalpur University, Jyoti Vihar, Burla, Sambalpur, Odisha 768019, India

**Keywords:** Wetlands, Cyanobacteria, Eutrophication, Industrialization, Pollution

## Abstract

**Background:**

Cyanobacteria are common components of phytoplankton communities in most freshwater ecosystems. Proliferations of cyanobacteria are often caused by high nutrient loading, and as such can serve as indicators of declining water quality. Massive industrialization in developing countries, like India, has polluted fresh water bodies, including wetlands. Many industries directly discard their effluents to nearby water sources without treatment. In the Sambalpur District of India effluents reach the reservoir of the worlds largest earthen dam i.e Hirakud Dam. This study examines cyanobacteria communities in the wetlands of Sambalpur District, Odisha, India, including areas subjected to industrial pollution.

**Result & Discussion:**

The genera *Anabaena, Oscillatoria, Chroococcus, Phormidium* were dominant genera of polluted wetlands of Sambalpur districts. A positive correlation was found between total cyanobacterial species and dissolved oxygen levels, but cyanobacterial diversity was inversely related to BOD, COD, TSS, and TDS. High dissolved oxygen content was also associated with regions of lower cyanobacteria biomass.

**Conclusion:**

Cyanobacterial abundance was positively correlated to content of oxidisable organic matter, but negatively correlated to species diversity. Lower dissolved oxygen was correlated to decreased diversity and increased dominance by *Anabaena, Oscillatoria, Chroococcus, Phormidium* species, observed in regions characterized by deteriorated water quality.

## Background

**Figure 1:**
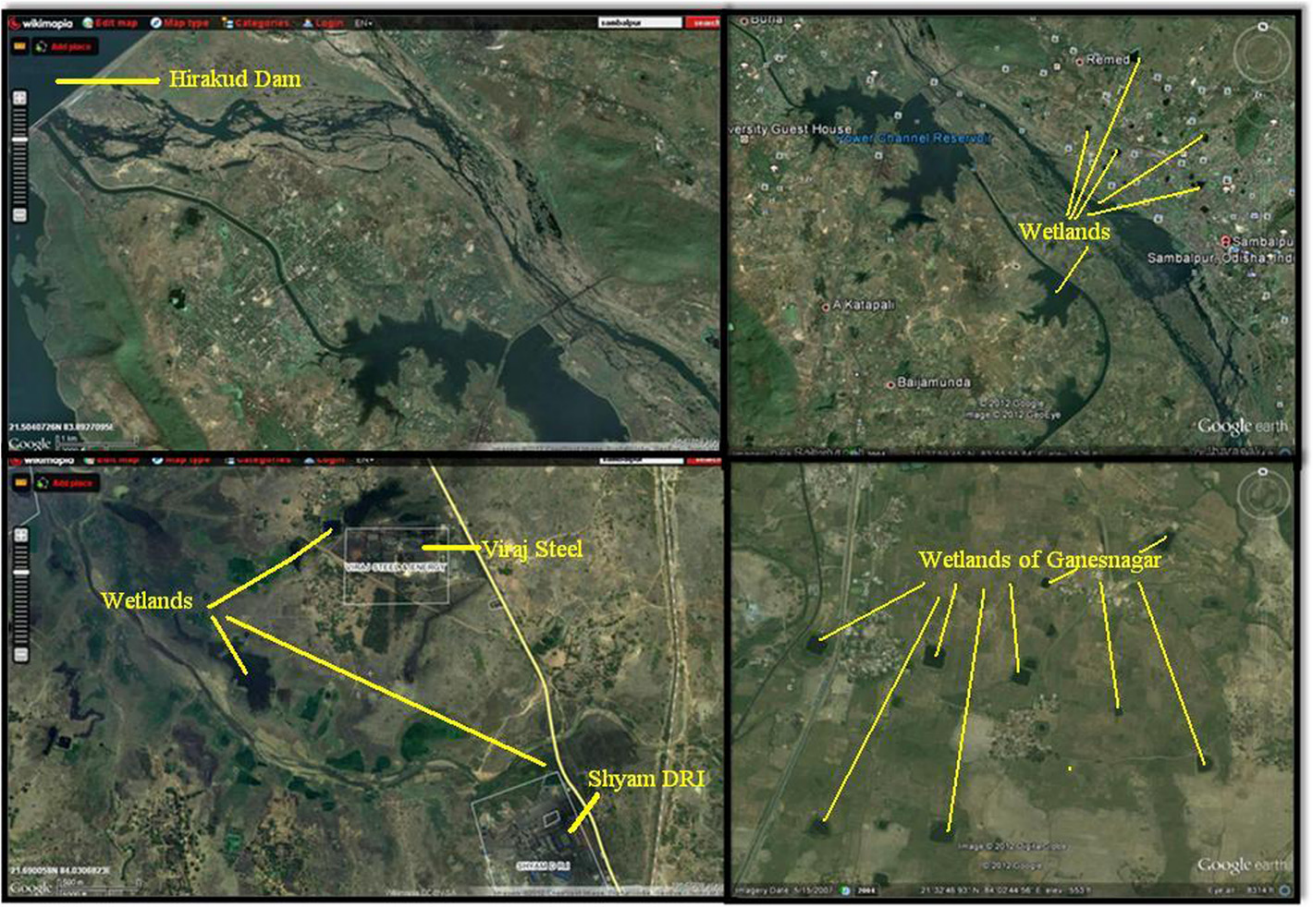
**Different wetlands of Sambalpur Districts and nearby area of Hirakud Dam.** Photo taken from Wikimapia (left) and Google earth (right).

Wetlands support a wide array of flora and fauna and deliver many ecological, climatic and societal functions. Scientists often refer to wetlands as the “kidneys” of the earth. However, many wetlands are subjected to urban and industrial pollution which disturb the aquatic ecosystem. Sambalpur is one of the main cultural and business centres of Odisha, India. It lies between 20°30′-22°30′N latitude and 83°E-85°1′longitudes with a total geographical area of 6,698 km^2^ Figure 1. The Hirakud reservoir in Sambalpur was built primarily for hydropower generation, but is also used for irrigation, fisheries and drinking water. Sambalpur contains various freshwater ecosystem types, including lakes, reservoirs, ponds and wetlands. Cyanobacteria have been shown to be key primary producers at the base of the food web of many of these types of systems, i.e. freshwater (Muthukumar et al., [1], paddy fields (Bhattacharyya et al., [2], soils (Adhikary [3], desert (Bhatnagar et al., [4], temple (Deepa et al., [5] estuaries (Palleyi, [6], hotspring and marine hypersaline (Aharon et al., [7], Komarek et al., [8]. However, large proliferations of cyanobacteria are often caused by high nutrient loading, and therefore can be indicators of declining water quality (Rivas et al., [9]; Garcia et al., [10], which can be associated with a range of problems, such as low oxygen levels and the production of algal toxins (Komarek, [11]. Anthropogenically-driven increase in eutrophication and pollution have led to increases in the frequency and intensity of cyanobacteria blooms in ecosystems world-wide (Hecky [12]; Rivas et al., [9]; Garcia et al., [10].

This investigation focused on determining cyanobacterial composition and diversity in wetland ecosystems under the influence of industrial pollution in Sambalpur, India. Little information is available on cyanobacteria in wetlands of this region. In Orissa many researchers (Ghadai et al., [13]; Dey et al., [14,15]; Prasanna and Nayak [16]; Sahu [17]; Adhikary [3] have focused on cyanobacteria in rice fields. Dey and Bastia [15]; Dash et al., [18] have studied algal flora of Simlipal biosphere. In the last five years, about 40 iron factories have been established in Sambalpur and its neighboring Jharsuguda district. In addition, a Super-thermal power plant, a paper mill and several cement factories are located in these two districts. Open cast coal mines of the Mahanadi coal fields are located in Western Orissa which includes Sambalpur, Jharsuguda and Sundargarh districts. Many of these industries discharge untreated water into local channels which drain into Hirakud reservoir. The municipal wastes of cities of this region also mix into the Mahanadi, the main river of this region. This study was carried out to provide important missing data needed to define algal flora within impacted wetland ecosystems of this region Das [19].

## Results and discussion

During the present investigation water samples were collected from 10 sites in wetland and stagnant water bodies associated with different industrial applications. The physicochemical properties of water of all site are described in Table 1 including pH, total suspended solids (TSS), total dissolved dolids (TDS), dissolved oxygen (DO), biological oxygen demand (BOD), chemical oxygen demand (COD), phosphate (PO_4_) and nitrate (NO_3_) content. In all the study sites, pH of water was in the alkaline range of 7.13 to 8.21. The relationship between alkaline conditions and cyanobacteria has been previously noted by several researchers Nayak and Prasanna [20]; Verma and Mohanty [21]. Mean TSS concentrations ranged from 14 Mg/l at Site 10 to 364 Mg/l at Site 5. Mean TDS ranged from 76 Mg/l at Site 10 to 840 Mg/l at Site 5. Mean dissolved oxygen levels ranged from 5.2 Mg/l at Site 5 to 11.2 Mg/l at Site 10. Mean BOD values ranged from 0.4 Mg/l Sites 4, 6, 9 and 10 to 1.8 Mg/l at Site 1. Mean COD values ranged from 7.4 Mg/l at Site 10 to 20.4 at Site 1. Mean nitrate (NO_3_) concentrations ranged from 9 Mg/l at Site 4 to 24 Mg/l at Site 1. Mean soluble reactive phosphorus (PO_4_) ranged from 7.5 at Site 10 to 18 Mg/l at Sites 1 and 9.

**Table 1:** Physiochemical properties of water at different location

**Sampli ng point**	**pH**	**TSS (Mg/l)**	**TDS (Mg/l)**	**DO (Mg/l)**	**BOD (Mg/l)**	**COD (Mg/l)**	**NO3 (Mg/l)**	**PO4 (Mg/l)**
Site1	8.12±0.04	312±14	562±12	6.2±0.5	1.8±0.02	20.4±1.1	24±3	18±3
Site2	7.13±0.2	32±4.5	174±10.5	8.4±1.12	0.6±0.03	10.2±2	16±2.2	12±2
Site3	7.22±0.02	68±6	150±25	7.5±0.04	0.6±0.02	13.2±1.2	18±0.6	14±1.2
Site4	7.51±0.06	41±2.6	132±11	9.1±0.15	0.4±0.1	8.1±0.06	9±1.2	8±0.08
Site5	7.92±0.04	364±12	840±15	5.2±0.22	1.6±0.14	18.6±0.18	22±1.06	34±1.8
Site6	7.32±0.12	22±1.6	160±6	8.8±0.3	0.4±0.06	9.3±0.3	18±1.4	16±0.8
Site7	7.91±0.05	180±5.6	241±4	7.2±0.4	0.6±0.01	8.2±0.21	12±2.04	10±1
Site8	8.21±0.11	62±2	188±6	8.0±0.6	0.9±0.3	10.4±0.06	14±0.8	15±2
Site9	7.98±0.03	170±2.8	364±11	7.7±0.02	0.4±0.05	9.2±0.06	16±0.8	18±2.0
Site10	7.60±0.04	14±1.2	72±6	11.2±0.3	0.4±0.16	7.4±0.6	22±1.0	7±0.06

Site1: Paddy Field water near SHYAM DRI Site2: Pond water Near Aryan Ispat and steel Pvt. Limited. Site3: Nearby wet lands of Viraj Steel Pvt. Ltd Site4: Stagnant water near Kherual bridge of M/s Bhusan Power and Steel Ltd. Site5: Paddy Field water near M/s Bhusan Power and Steel Ltd. Site6: Pond water near the Rengali WESCO office Site7: Water from local nallah near Debaipali Site8: Pond water of Sason village Site9: Pond water near Ganesh Nagar. Site 10. Guest House pond and nearby wetlands of Sambalpur University.

**Figure 2:**
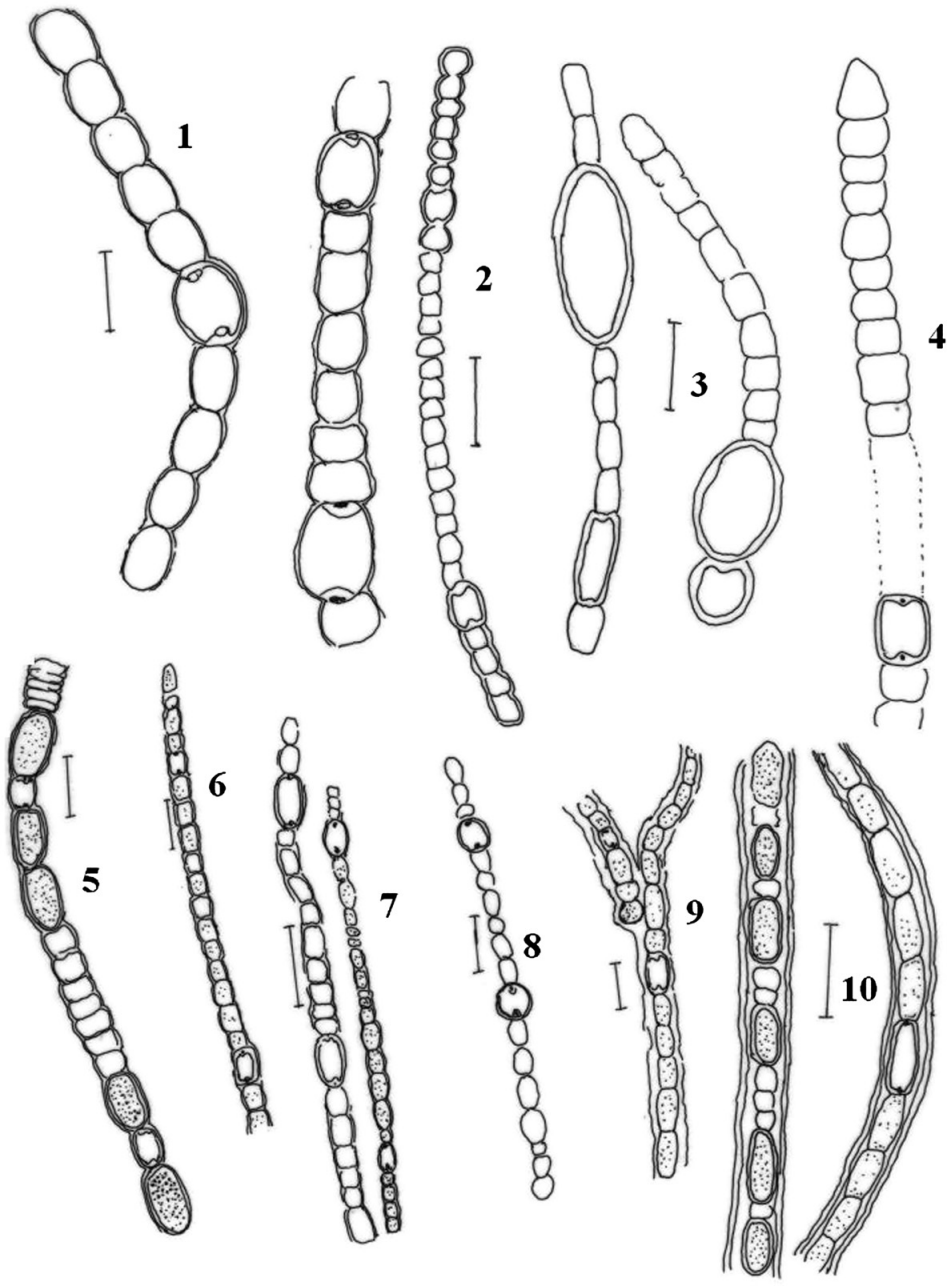
**Camera Lucida diagram, of cyanobacterial species *1*. ***Anabaena azollae****2. *** Anabaena fertilissima 3. Anabaena marcospora 4. Anabaena orientalis ***5. ****Anabaena oscillarioides****6. ****Anabaena sphaerica****7. ****Anabaena variabilis****8. ****Anabaena iyengarri****9. ****Aulosira fertilissima****10. ****Aulosira bombayensis*. Scale bar 10 μm.

**Figure 3:**
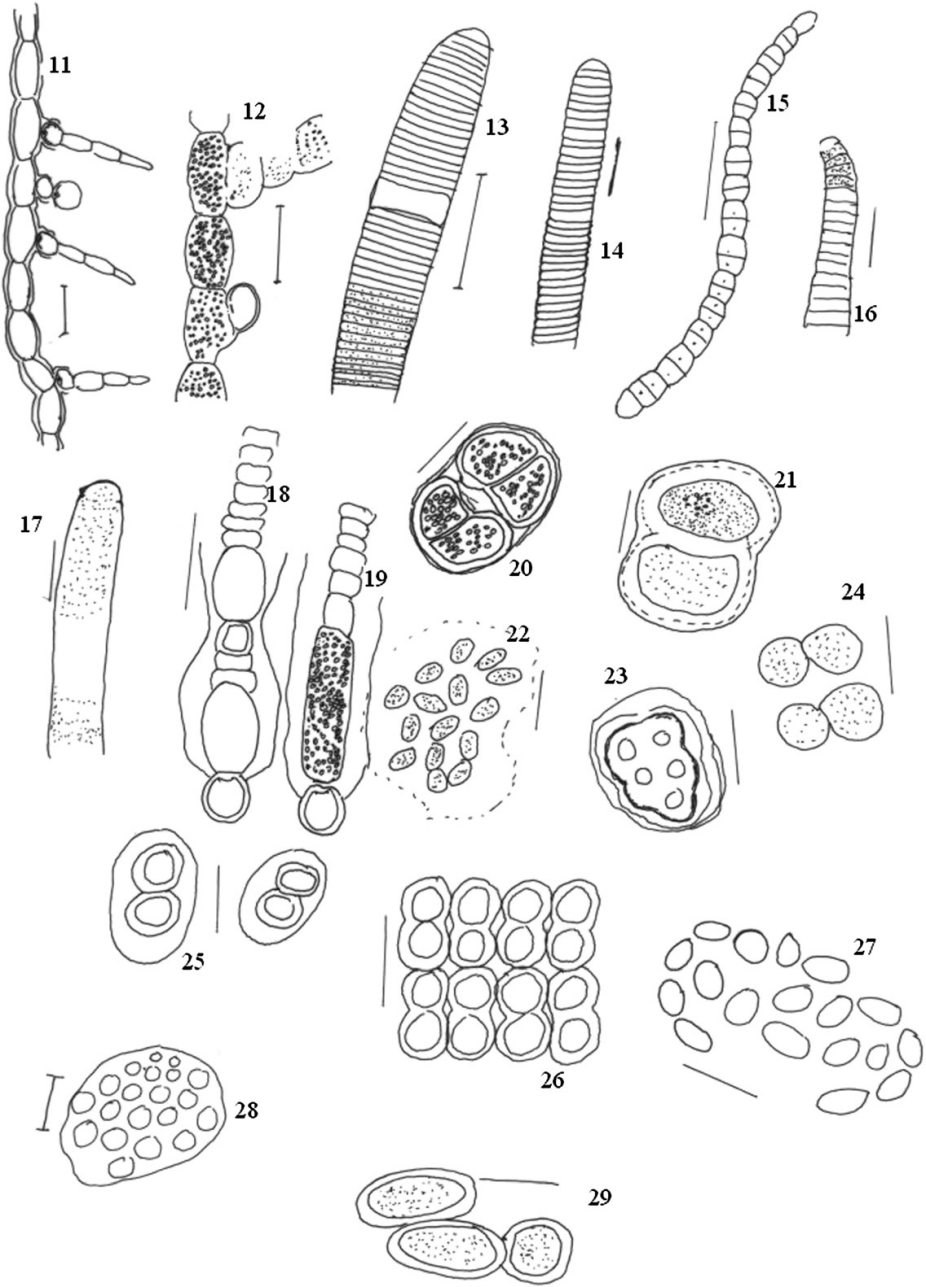
**Camera Lucida diagram, of cyanobacterial species *11. ****Nostochopsis radians****12. ****Nostochopsis lobatus****13. ****Oscillatoria simplicissima****14. ****Oscillatoria jenesis****15. ****Oscillatoria sp.****16. ****Oscillatoria princeps****17. ****Oscillatoria limosa****18. ****Gloeotrichia sp.****19. ****Gloeotrichia natans****20. ****Chroocococcus tanex****21. ****Chroococcus limneticus****22. ****Gloeocapsa gelatinosa****23. ****Gloeocapsa sp.****24. ****Chroococcus turgidus****25. ****Gloeocapsa sp.****26. ****Merismopodia sp.****27. ****Aphanothece conferta****28. ****Coelosphaerium sp.****29. ****Gloeothece rupestris.* Scale bar 10 μm.

**Figure 4:**
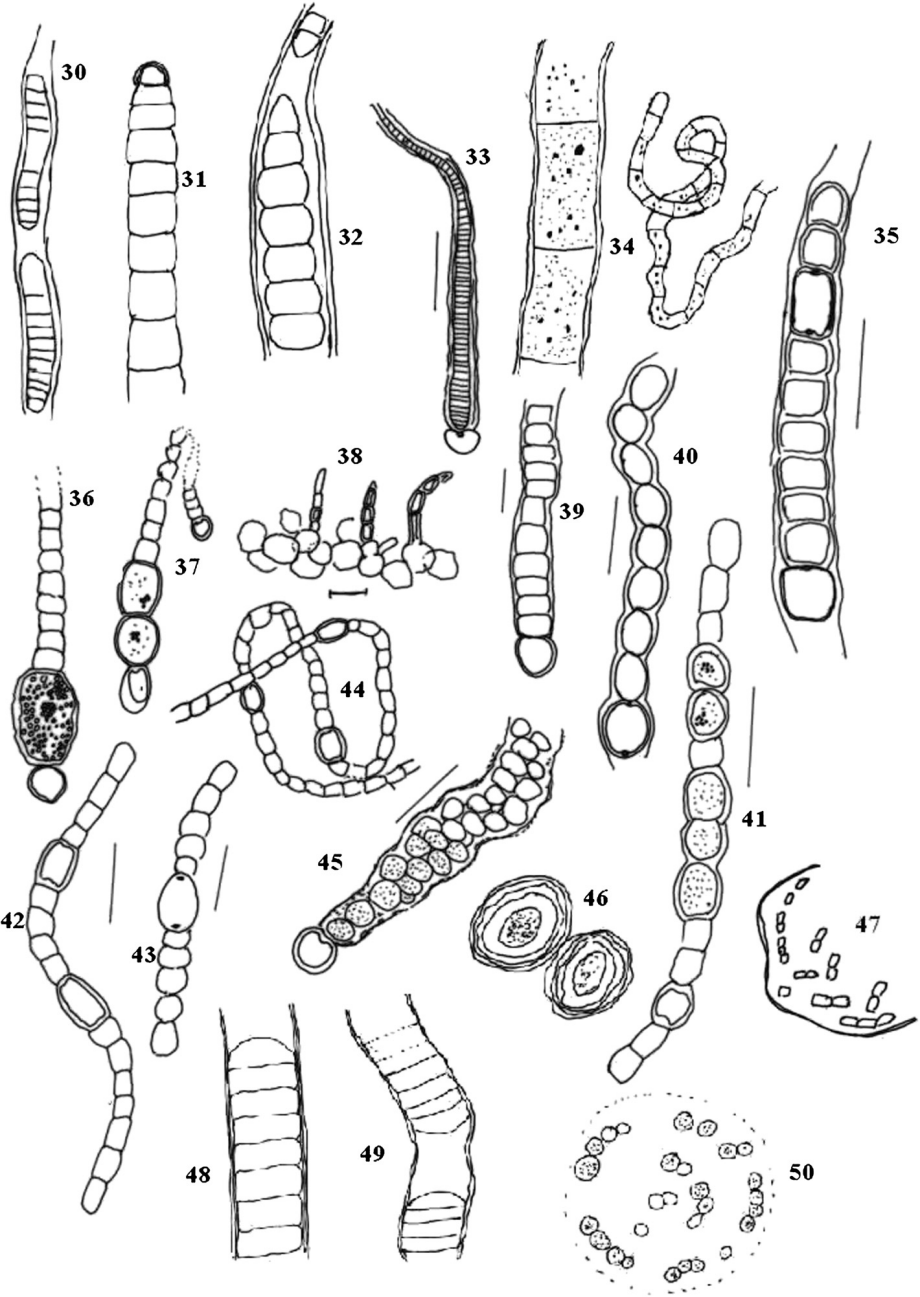
**Camera Lucida diagram, of cyanobacterial species *30.*** Phormidium purpurascans ***31. ****Phormidium****32. ****Phormidium****33. ****Calothrix braunii****34. *** Phormidium sp. ***35. *** Microchaete aequalis ***36. *** Cylindrospermum muscicola ***37. *** Cylindrospermum sp. ***38. *** Fischerella muscicola ***39. ****Microchaete sp.****40. ****Microchaete sp.****41. ****Nostoc spongiforme****42. *** Nostoc commune ***43. *** Nostoc muscorum ***44. *** Nostoc carneum ***45. *** Nostoc punctiforme ***46. ****Aphanothece microscopica****47. ****Aphanothece microscopic****48. ****Lyngbya sp.****49. ****Lyngbya sp.****50. ****Chroococcus sp.* Scale bar 10 μm.

**Figure 5:**
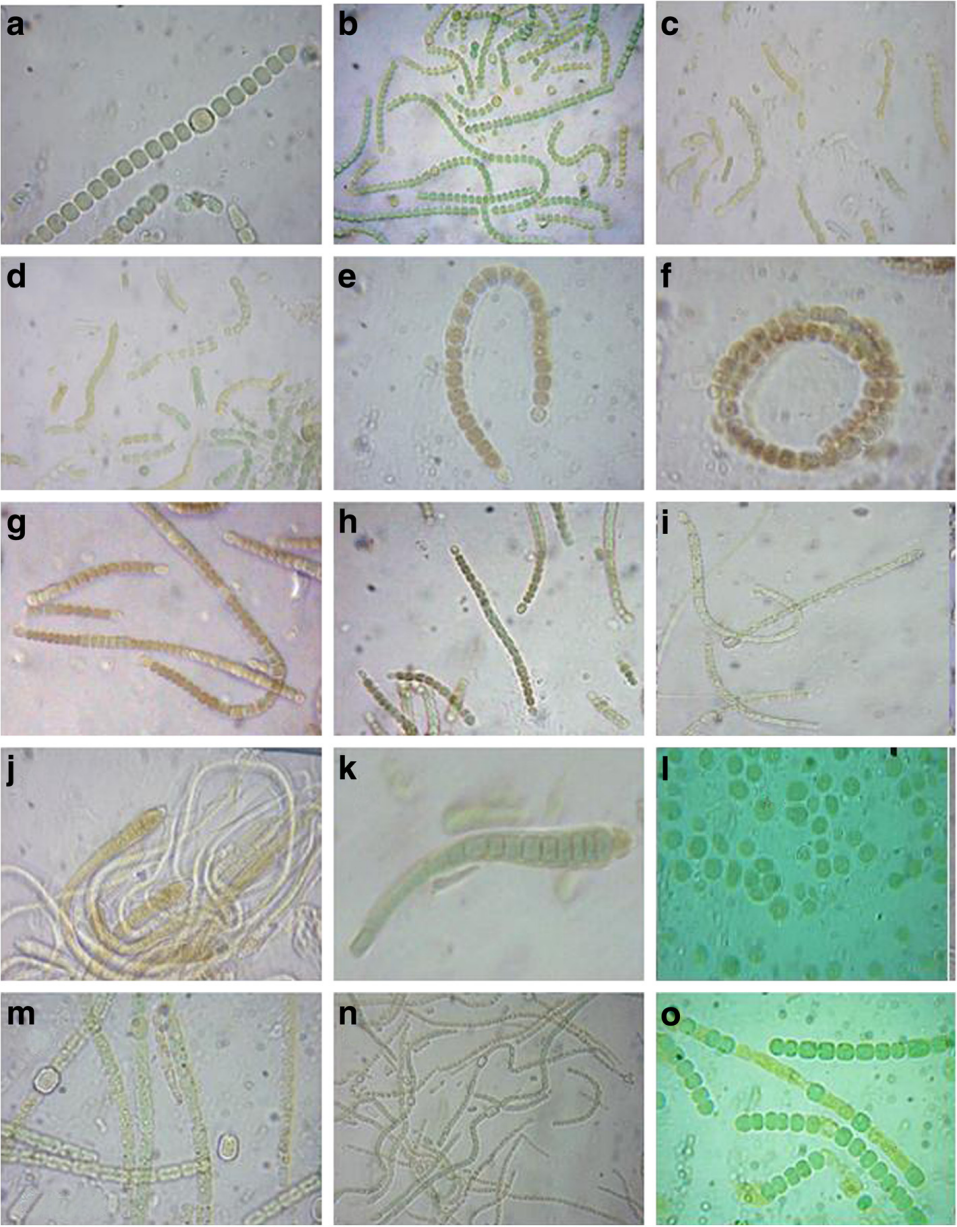
**Cyanobacterial strains under microscope: a.***Nostoc spongiforme;***b. ***Nostoc spongiforme;***c. ***Nostoc sp.;***d. ***Nostoc sp.;***e. ***Anabaenopsis sp.;***f. ***Anabaena circularis;***g. ***unidentified;***h. ***Anabaena roxburgii;***i. ***Anabaena variabilis***j. ***Calothrix sp.;***k. ***Microchaetae sp.;***l. ***Gleocapsa sp.;***m. ***Nostoc muscorum;***n. ***Anabaena sp.;***o. ***Anabaena sp.*

Cyanobacterial strains were observed under a light microscope and further described with the aid of Camera Lucida, which helped to define taxonomically important characteristics, such as shape of vegetative cell, presence of heterocysts and akinetes (Figures 2, 3, 4, and 5). Results showed that Nostocales, and Chroococcales are major groups in the wetlands of this region. Over the study period, a total of 55 species and 20 different cyanobacteria genera were isolated and documented (Table 2) of which 30 were heterocystous forms and 24 were non-heterocystous. Relative abundance percentages of individual species are shown in Table 2. Oscillatoriales, Nostocales and Chroococcales species showed the highest relative abundance values. *Chroococcus turgidus and Nostoc punctiforme* were found in almost all sites except Site 3 and Site 5 respectively. Two species of *Haplosiphon* were only found in samples from the guest house pond (control), although it is one of the major species observed in rice paddy fields by Das [19]. In terms of frequency of occurrence the genera *Anabaena, Chroococcus, Gloeocapsa, Nostoc, Oscillatoria and Phormidium* were observed at all sites, while at the other end of the spectrum *Coelospherium* and *Fischerella* were only observed in 33% (Table 3). Presence of *Anabaena*, *Oscillatoria* and *Chroococcus* indicate the polluted values of wetlands.

**Table 2:** Diversity of Cyanobacteria in different wetlands of Sambalpur

**Name of species**	**Site1**	**Site2**	**Site3**	**Site4**	**Site5**	**Site6**	**Site7**	**Site8**	**Site9**	**Site 10**	**FO**	**RF**	**RD**	**RA**
*Anabaena azolae*	+	+	+	+	+	-	+	-	+	+	80	3.08	5.81	3.60
*Anabaena fertilissima*	-	+	+	-	-	+	-	-	-	+	40	1.54	1.33	1.65
*Anabaena iyengani*	+	+	-	+	-	-	+	+	+	-	60	2.31	1.66	1.37
*Anabaena marcospora*	+	-	+	-	-	-	+	-	-	+	40	1.54	0.83	1.03
*Anabaena orientalis*	-	+	+	-	-	+	+	-	+	-	50	1.92	1.33	1.32
*Anabaena oscillarioides*	+	-	-	+	+	+	-	+	-	+	60	2.31	2.49	2.06
*Anabaena sphaerica*	-	-	+	+	-	-	-	-	-	-	20	0.77	1.00	2.47
*Anabaena variabilis*	-	+	-	-	-	+	+	-	-	+	40	1.54	1.66	2.06
*Aphanocapsa grevillei*	-	-	-	-	-	+	-	+	+	-	30	1.15	0.83	1.37
*Aphanocapsa sp*		+		+			+			-	30	1.15	0.66	1.10
*Aphanocapsa sp*	+	-	+	-	-	+	+	-	+	+	60	2.31	1.99	1.65
*Aphanothece conferta*	+	+	-	+	-	+	-	+	+	-	60	2.31	3.32	2.74
*A*. *microscopica*	+	+	+	-	+	-	-	+	+	-	60	2.31	1.33	1.10
*Aulosira bombayensis*	-	-	-	+	-	+	-	-	-	+	30	1.15	1.00	1.65
*Aulosira fertilisima*	+	+	-	-	-	-	+	+	+	+	60	2.31	2.16	1.78
*Calothrix braunii*	+	-	+	-	+	-	-	-	+	+	50	1.92	1.16	1.15
*Calothrix clavatoides*	+	-	-	+	-	+	-	+	-	+	50	1.92	0.83	0.82
*Calothrix javanica*	-	+	-	-	-	-	-	+	-	+	30	1.15	0.83	1.37
*Calothrix linearis*	+	+	-	-	-	+	-	-	-	+	40	1.54	1.99	2.47
*Calothrix parientina*	-	-	+	+	-	+	+	-	-	-	40	1.54	1.33	1.65
*Chrococcus limneticus*	+	-	-	-	+	+	-	+	+	-	50	1.92	1.33	1.32
*Chrococcus turgidus*	+	+	+	+	-	+	+	+	+	+	90	3.46	4.49	2.47
*Chrocococcus tanex*	+	-	+	-	+	-	-	-	+	-	40	1.54	2.33	2.88
*Coleosphaerium sp*	+	-	-	-	-	+	-	-	+	-	30	1.15	0.66	1.10
*Cylindrospermum sp*	-	-	+	+	-	-	-	-	+	+	40	1.54	0.83	1.03
*C*. *muscicola*	+	-	-	-	-	+	+	+	-	+	50	1.92	2.16	2.14
*Fischerella muscicola*	-	-	+	+	-	+	-	-	-	+	40	1.54	1.16	1.44
*Gleocapsa sp*	-	-	-	+	+	-	-	+	-	-	30	1.15	1.83	3.02
*Gleocapsa*		+				+	+	+	+	+	60	2.31	2.16	1.78
*Gleocapsa*	+	+	+	+	+	-	-	+	+	-	70	2.69	4.15	2.94
*Gleotheceae sp*	+	+	-	-	-	-	+	-	+	+	50	1.92	2.49	2.47
*Gleotrichia natans*	+	-	-	-	+	-	-	+	+	+	50	1.92	1.00	0.99
*Gleotrichia sp*	-	-	+	+	-	+	-	+	+	-	50	1.92	1.50	1.48
*Haplosiphon*	-	-	-	-	-	-	-	-	+	+	20	0.77	0.50	1.23
*Haplosiphon sp*	-	-	-	-	-	-	-	-	+	+	20	0.77	0.50	1.23
*Lyngbya sp*	+	-	+	+	+	-	-	-	-	+	50	1.92	1.50	1.48
*Lyngbya*	+	+	-	-	-	+	+	+	+	+	70	2.69	3.65	2.59
*Merismopedia eligans*	-	-	-	-	-	-	-	-	+	-	10	0.38	0.17	0.82
*Merismopodia sp*	-	+	-	+	-	+	-	+	-	-	40	1.54	1.83	2.26
*Microcheate sp*	+	-	-	+	+	-	-	+	-	-	40	1.54	2.16	2.67
*Microcheate aequalis*	-	+	-	-	-	+	-	+	-	-	30	1.15	1.16	1.92
*Nostoc carneum*	+	-	+	+	+	+	+	-	+	+	80	3.08	3.16	1.95
*Nostoc commune*	+	-	-	-	-	+	-	-	-	+	30	1.15	1.00	1.65
*Nostoc muscorum*	-	-	+	-	-	-	-	+	-	+	30	1.15	1.33	2.19
*Nostoc punctiformae*	+	+	+	+	-	+	+	+	+	+	90	3.46	2.16	1.19
*Nostoc spongiformae*	+	-	-	+	-	-	-	-	-	+	30	1.15	1.00	1.65
*Nostocopsis radians*	-	-	+	-	-	+	-	-	+	-	30	1.15	0.83	1.37
*Nostocopsis sp*	+	+	-	+	-	-	-	+	-	-	40	1.54	0.83	1.03
*Oscillatoria limosa*	+	-	+	-	+	+	-	-	-	+	50	1.92	3.49	3.45
*Oscillatoria princes*	+	+	-	+	-	+	-	+	+	+	70	2.69	3.82	2.70
*Oscillatoria sp*	+	+	-	+	-	+	+	-	-	+	60	2.31	2.66	2.19
*Oscillatoria sp*	+	-	+	-	-	-	-	+	+	-	40	1.54	0.83	1.03
*Phormidium sp*	+	+	+	-	+	+	+	+	-	+	80	3.08	3.49	2.16
*Phormodium*	+	-	-	+	+	+	+	+	-	-	60	2.31	2.82	2.33
*Phormodium*	-	+	+	+	-	+	-	-	-	+	50	1.92	1.50	1.48
*Total*	33	24	24	26	15	31	19	27	26	33				

+: present; -: not recorded.

**Table 3:** Major genera and their relative abundance in the sample

***Genus***	**Total no of species**	**Frequency of occurance**
*Anabaena*	8	100
*Chroococcus*	3	100
*Gloeocapsa*	3	100
*Nostoc*	5	100
*Oscillatoria*	4	100
*Phormidium*	3	100
*Aphanothece*	2	89
*Calothrix*	1	22
*Lyngbya*	2	89
*Aphanocapsa*	3	78
*Aulosira*	2	78
*Cylindrospermum*	2	78
*Gloeotrichia*	2	78
*Microchaete*	2	67
*Merismopodia*	2	56
*Gloeothece*	1	45
*Nostochopsis*	2	45
*Coelosphaerium*	1	33
*Fischerella*	1	33

Diversity index of cyanobacterial populations at the study sites were calculated using the Shannon-Wienner Method (1949) (Table 4). Sites 3, 4, 6, 8 and 10 had indices over 3 (Table 4). Sites 1, 2, 7 and 9 had indices between, 2 and 3, while Site 5 had the lowest value, i.e. 1.24. The two sites with the lowest diversity, Sites 1 and 5, also exhibited the lowest mean DO values and the highest mean TSS, TDS and COD values.

**Table 4:** Occurrence and distribution of cyanobacteria in various locations of Sambalpur, India along with Shannon-Wienner diversity index (H)

**Location**	**Total no of genera**	**Non heterocystous forms**	**Heterocystous forms**	**Shannon (H)**
Site 1	17	**18**	15	2.28
Site 2	14	**11**	**11**	**2.49**
Site 3	13	**10**	**12**	**3.01**
Site 4	15	**10**	**13**	**3.03**
Site 5	11	**10**	**5**	**1.24**
Site 6	17	**14**	**15**	**3.4**
Site 7	11	**7**	**10**	**2.82**
Site 8	16	**15**	**11**	**3.19**
Site 9	14	**7**	**9**	**2.87**
Site 10	17	**11**	**22**	**3.35**

**Figure 6:**
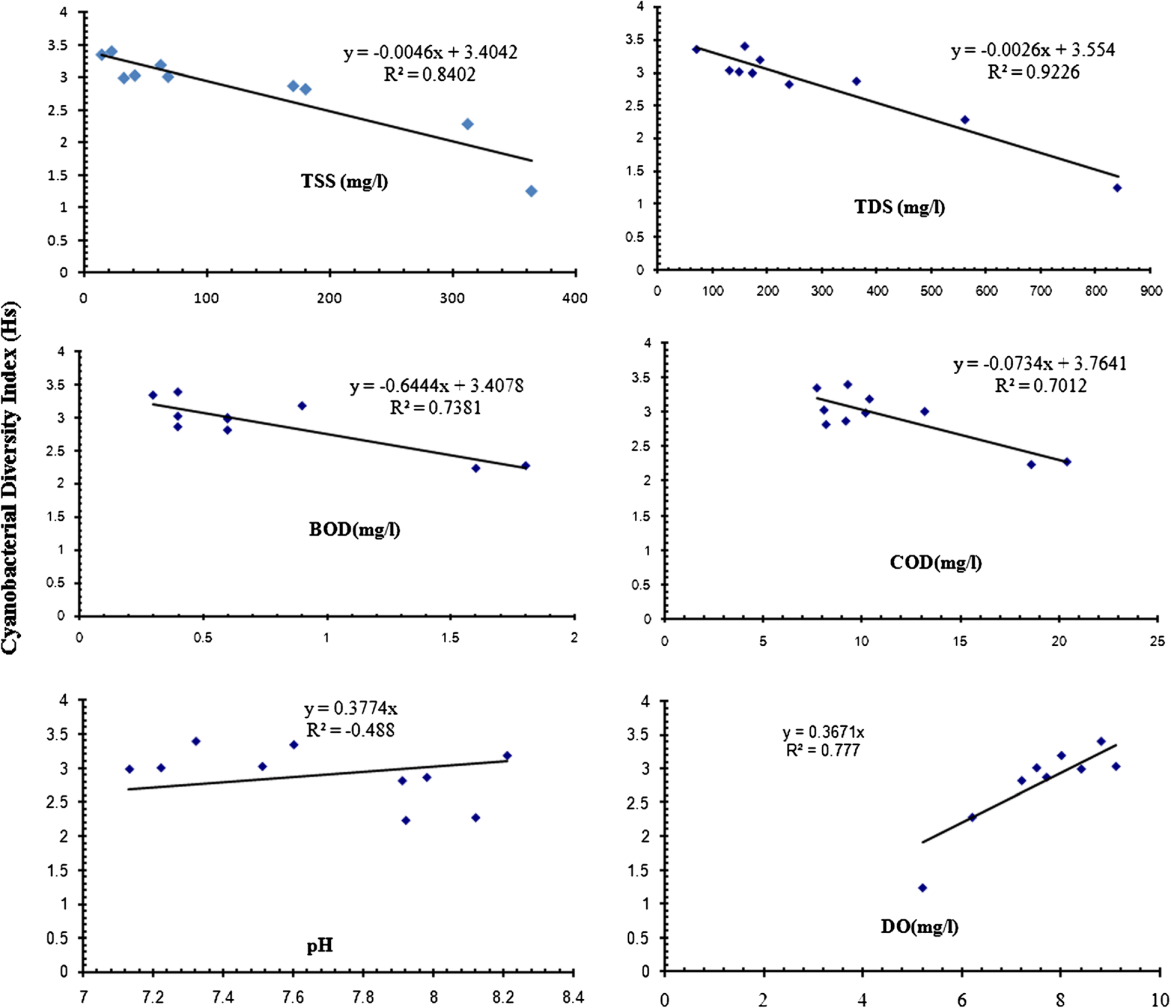
Correlation between Cyanobacterial diversity index and physicochemical parameter of water.

Correlation analysis (Table 5) of physicochemical properties of water samples and total cyanobacterial species (TCS) revealed a positive correlation (Figure 6) between dissolved oxygen and TCS (r=0.9385 p<0.01), which supports the findings of Muthukumar et al., [1]. In addition, the negative correlations between TCS and TSS, TDS, BOD, and COD, although not significant at the 0.01 level, further suggest that reduced water quality is associated with lower cyanobacterial diversity. By contrast, cyanobacterial abundance or biomass can increase in polluted systems associated with heavy nutrient loads. Kim et al., [22] found a positive correlation between prevalence of cyanobacteria and the levels of pollution in reservoir water, including the following species *Anabaena azollae, Anabaena oscillarioides, Aphanothece microscopic, Chroococcus limneticus, Chroococcus turgidus, Chroococcus tenax, Gloeocapsa, Lyngbya, Oscillatoria, Phormidium.* The crucial role in physicochemical parameters in defining algal community composition and abundance in agricultural and wetland ecosystems of tropical and temperate regions has been examined by a number of researchers (Kohler, [23]; Chellappa et al., [24]; Prasanna et al., [25].

**Table 5:** Correlation co-efficient analysis of physicochemical properties of water and total cyanobacterial species (TCS)

	**pH**	**TSS**	**TDS**	**DO**	**BOD**	**COD**	**NO**_**3**_	**PO**_**4**_	**TCS**
**pH**	1								
**TSS**	0.620829	1							
**TDS**	0.513552	0.947356*	1						
**DO**	−0.51899	−0.943732	−0.908277	1					
**BOD**	0.535549	0.828902*	0.827283	−0.844629	1				
**COD**	0.321332	0.783196*	0.804691	−0.830509	0.93978*	1			
**NO**_**3**_	0.158125	0.640212	0.703164	−0.709126	0.75499	0.880077*	1		
**PO**_**4**_	0.338464	0.73854	0.893818*	−0.784829	0.66081	0.694318	0.717146	1	
**TCS**	−0.01215	−0.331114	−0.360616	0.938492*	−0.05977	−0.00745	0.130953	−0.39107	1

* significant ≤ 0.01.

Rapid cyanobacterial growth in the micro aerophilic condition has been observed by Stewart and Parsons [26]. Rai and Kumar [27] did not find heterocystous cyanobacteria in polluted water, although in our study various heterocystous species, including the genera *Anabaena, and Nostoc,* were commonly present. Presence of *Anabaena,* and some other blooming cyanobacteria have been linked to low DO content in eutrophic waterbodies (Moss, [28]; Mbonde et al., [29]. In terms of non-heterocystous taxa, *Oscillatoria* has been found to be tolerant to polluted water (Singh and Saxena [30]. Our observation of the widespread presence of the non-heterocystous genera *Oscillatoria, Phormidium, Gloeocapsa,* and *Chroococcus* corresponds to the findings of other wetland systems (Palmer, [31], 1980; Dubey et.al., [32] and Ghadai et al., [13].

## Conclusions

Cyanobacteria are important primary producers at the base of the microbial food web in many aquatic environment. The composition and diversity of cyanobacterial communities can provide insights into changes in water quality. The present study provides a baseline of information on cyanobacterial composition associated with tropical wetland habitats under the influence of significant industrial development. This information can also be used to identify candidate species for use in bioremediation of industrial waste, since the species found in these systems are adapted to the stresses imposed by the components of the waste.

## Methods

### Study site

**Figure 7:**
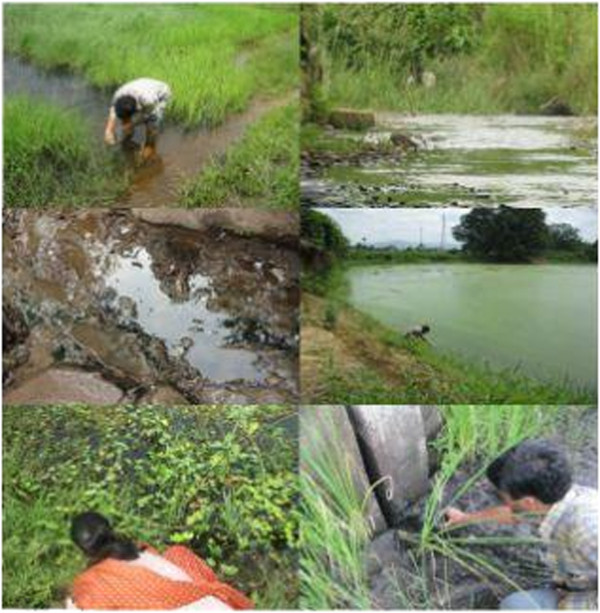
Water sample collected from different wetland of Sambalpur, Odisha, India.

The study was conducted in different wetlands of Sambalpur districts, Odisha. Nine sampling sites (Figures 1 and 7) were selected from different industrial regions. A Guest house pond at Sambalpur University was selected as a site not directly influenced by industrial development. The sites are as follows: Site1, Paddy field water near ShyamDRI; Site2, Pond water near Aryan Ispat and steel Pvt. Limited; Site3, In wetland near Viraj Steel Pvt. Ltd; Site4, Stagnant water pond near Kherual bridge of M/s Bhusan Power and Steel Ltd.; Site5, Paddy field near M/s Bhusan Power and Steel Ltd.; Site6, Pond near the Rengali WESCO office; Site7, Pond near Debaipali; Site8, Pond near Sason village; Site 9, Pond near Ganesh Nagar; Site 10, Guest House pond at Sambalpur University.

Surface grab water samples were collected in bottles from each site for microscopic analysis and isolation of cyanobacterial strains.

### Collection of water sample

Water sample are collected in 250 ml air tight plastic jars. A few rocks from wetlands were collected to examine attached cyanobacteria.

### Physicochemical properties of water

The physicochemical analyses of the water samples were carried out by using standard methods (APHA, [33] and Trivedy and Goel [34].

### Isolation and enumeration of cyanobacteria

One ml water samples were added to agar plates made with 25 ml of sterilized BG ± 11 medium in petri dishes (Rippka et al., [35] The dishes were kept under 7.5 W/m^2^ light intensity at 25±0.5^o^C in a culture room. After 10-12 days of incubation, algal colonies appeared on the agar plates. The number of colonies of each species were recorded (CFU), after microscopic observation. Colonies were then isolated and spread on to fresh agar plates. After development, colonies appearing in agar plates were examined microscopically and transferred to agar slants. This process was repeated until individual pure colonies were obtained free from any contamination.

### Microscopic analysis

Isolated cyanobacterial strains were observed under microscope MLX-TR. For morphometric analysis Camera Lucida Drawings was done and data related to trichome shape, filament colour, akinete and heterocyst shape, size, position, number recorded. Identification of cyanobacteria was done using the keys given by Desikachary [36] and Komarek and Anagnostidis [37,38].

### Data analysis

a. Frequency of Occurance:

$$\begin{array}{ll} \;\mathrm{FO}= & \frac{\mathrm{Number}\;\mathrm{of}\;\mathrm{sample}\;\mathrm{containing}\;\mathrm{the}\;\mathrm{species}}{\mathrm{Total}\;\mathrm{no}\;\mathrm{sample}\;\mathrm{examined}}\\ & \times 100 \end{array}$$

b. Relative Frequency:

$$\begin{array}{ll} \;\mathrm{RF}= & \frac{\mathrm{Number}\;\mathrm{of}\;\mathrm{sample}\;\mathrm{containing}\;a\;\mathrm{species}}{\mathrm{Total}\;\mathrm{no}\;\mathrm{of}\;\mathrm{occurance}\;\mathrm{of}\;\mathrm{all}\;\mathrm{the}\;\mathrm{species}}\\ & \times 100 \end{array}$$

c. Relative Density:

$$\begin{array}{ll} \;\mathrm{RD}= & \frac{\mathrm{Number}\;\mathrm{of}\;\mathrm{CFU}\;\mathrm{of}\;a\;\mathrm{species}\;\mathrm{in}\;\mathrm{all}\;\mathrm{samples}}{\mathrm{Total}\;\mathrm{no}\;\mathrm{of}\;\mathrm{CFU}\;\mathrm{all}\;\mathrm{the}\;\mathrm{species}\;\mathrm{in}\;\mathrm{all}\;\mathrm{the}\;\mathrm{samples}}\\ & \times 100 \end{array}$$

d. Relative abundance:

The relative abundance of a particular cyanobacteria type was calculated by employing the formula:

$$\begin{array}{ll} \;\mathrm{RA}\;= & \;\frac{\mathrm{Number}\;\mathrm{of}\;\mathrm{samples}\;\mathrm{containing}\;\mathrm{the}\;\mathrm{species}}{\mathrm{Total}\;\mathrm{no}\;\mathrm{of}\;\mathrm{occurance}\;\mathrm{of}\;\mathrm{all}\;\mathrm{the}\;\mathrm{species}}\\ & \times 100 \end{array}$$

e. Diversity index: Cyanobacterial diversity in different sites has been calculated by Shannon’s Diversity index (Shannon Wienner [39] as per the following formula

$$\mathrm{Hs}=-\overset{S}{\underset{i=1}{\sum }}\left(\mathrm{Pi}\right)\left(\mathrm{lnPi}\right)$$

Where,

Hs- diversity in the sample S species or kinds

S- the Number of Species in the Sample

Pi- relative abundance of i^th^ species or Kind measures,n_i_/N

N- total no of individuals of all kinds

n_i_- no of individual of i^th^ species

f. Correlation coefficient were calculated as per [40], using Microsoft Excel 2007 Package and analysed for their significant using Pearson’s table

## Abbreviations

TSS: Total suspended solids; TDS: Total dissolve solids; DO: Dissolve oxygen; BOD: Biological oxygen demand; COD: Chemical oxygen demand; TCS: Total cyanobacterial species; CFU: Colony forming unit.

## Competing interests

The authors declare that they have no competing interests.

## Authors’ contributions

PRD performed the experiments, SB calculated results, prepared the Table, Graph, Diagram, Statistical analysis and composed the Draft for manuscript in consultation with BN. BN designed the experiment, SB analysed and interpreted data and result, modified the manuscript in final form. BN planned the project, was involved in acquisition of funds. BN selects the sites of sample collections. All the authors read and approved the final manuscript.
